# Association between diabetes mellitus and risk of Parkinson's disease: A prisma‐compliant meta‐analysis

**DOI:** 10.1002/brb3.2082

**Published:** 2021-07-21

**Authors:** Wei Liu, Jianfeng Tang

**Affiliations:** ^1^ Department of Metabolism and Endocrinology Yongzhou Central Hospital Yongzhou China

**Keywords:** diabetes mellitus, meta‐analysis, Parkinson's disease

## Abstract

**Background:**

Previous studies showed inconsistent results regarding associations between diabetes mellitus (DM) and risk of Parkinson's disease (PD). The study aimed to make a meta‐analysis to clarify whether DM is a risk factor for PD.

**Methods:**

We searched for articles regarding the effect of DM on risk of PD and published before July 2020 with search terms as follows: (“diabetes mellitus” OR “diabetes”) AND (“Parkinson's disease” OR “PD”) in the following databases: PubMed, Web of Science, MEDLINE, EMBASE, and Google Scholar. We used STATA 12.0 software to compute multivariate odds ratio (OR) or relative risk (RR) and 95% confidence intervals (CI) regarding the association between DM and risk of PD.

**Results:**

The present study finally included 7 case–control studies (including 26,654 PD patients) and 9 cohort studies (including 3,819,006 DM patients) exploring the association between DM and risk of PD. The meta‐analysis indicated that DM was related to elevated risk of PD (OR/RR = 1.15, 95% CI 1.03–1.28, *I* ^2^ = 92.4%, *p* < .001). Subgroup study showed that DM was associated with higher risk of PD in cohort studies (RR = 1.29, 95% CI 1.15–1.45, *I* ^2^ = 93.9%, *p* < .001), whereas no significant association was indicated between DM and risk of PD in case–control studies (OR = 0.74, 95% CI 0.51–1.09, *I* ^2^ = 82.3%, *p* < .001). Sensitivity analysis showed no changes in the direction of effect when any one study was excluded from all meta‐analyses. In addition, Begg's test, Egger's test, and funnel plot showed no significant risks of publication bias.

**Conclusion:**

In conclusion, we have tried to determine whether prior onset of DM may contribute to the risk of developing PD. More and more large‐scale prospective studies should be conducted to evaluate the relationship between DM and PD.

## Graphical abstract

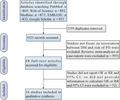

## 1 INTRODUCTION

Parkinson's disease (PD) is a progressive neurodegenerative disease that affects 1% of the population aged 60 or above (Tysnes & Storstein, 2017). PD is a common nervous system disease characterized by tremor and bradykinesia/akinesia (Hayes, 2019). The prevalence of PD is 1‰–2‰ throughout the world and increasing with age (Mak & Wong‐Yu, 2019). It has been confirmed that PD is associated with the decrease in the neurotransmitter dopamine (Fredericks et al., 2017). Up to now, the etiology of PD is not clear. Multiple genetic, environmental factors, such as glucocerebrosidase gene mutations, pesticide exposure, and their interactions, seem to be associated with PD (Delamarre & Meissner, 2017; Kalia & Lang, 2015).

Diabetes mellitus (DM) is a complex metabolic disorder with a prevalence rate of 1 per 11 of the adult population globally, and associated with multiple systems and organs (Zaccardi et al., 2016; Zheng et al., 2018). About 400 million people are affected by DM worldwide, 90% of whom are type 2 diabetes mellitus (T2DM; Tsilidis et al., 2015). Several studies showed the mutual influence between insulin and dopamine (Garcia Barrado et al., 2015; Jones et al., 2017; Patel et al., 2019). However, these studies on the association between PD and DM showed inconsistent results (Cereda et al., 2011; Lu et al., 2014). De Pablo‐Fernandez et al., (2018) reported an increased rate of subsequent PD following DM in a large cohort study, whereas Simon et al., (2007) reported that PD risk is not significantly related to history of diabetes with a large prospective study. Considering the heavy burden of PD and DM for public health, studies on the association between PD and DM are of significance. Thus, we systematically reviewed the published literature regarding the relationship between DM and risk of PD, and conducted this meta‐analysis to clarify whether DM is a risk factor for PD.

## 2 METHODS

The study was performed according to the Preferred Reporting Items for Systematic reviews and Meta‐Analysis (PRISMA) guideline (Moher et al., 2009).

### 2.1 Search strategy and selection criteria

We searched for articles published before July 2020 with search terms as follows: (“diabetes mellitus” OR “diabetes”) AND (“Parkinson's disease” OR “PD”) in the following databases: PubMed, Web of Science, MEDLINE, EMBASE, and Google Scholar.

After excluding duplicates, 1,021 studies were included. Selection criteria were shown as follows: (a) studies that provided sufficient information regarding odds ratio (OR) or relative risk (RR) and 95% confidence intervals (CI) associated with DM and risks of PD; and (b) studies whose OR or RR and 95% CI could be calculated from the data in the studies. In addition, included studies could not be reviews, meta‐analyses, or case reports. All the abstracts and full texts were read independently by two investigators (Wei Liu and Jianfeng Tang).

### 2.2 Data extraction

Two investigators (Wei Liu and Jianfeng Tang) independently used an Excel file to extract data from finally included studies. These data included author, publication year, study design, study location, sample sizes, gender of participants, event for analysis, adjustment factors, and results.

### 2.3 Meta‐analysis

We used STATA 12.0 software to compute the results extracted from finally included studies. We used *Q* test and inconsistency index (*I* ^2^) to assess heterogeneities between studies. When the heterogeneity was high (*p* value for *Q* test ≤ 0.05 and *I* ^2^ ≥ 50%), random‐effects models were used to compute the results; when the heterogeneity was low (*p* value for *Q* test > 0.05 and *I* ^2^ < 50%), fixed‐effects models were used to compute the results. Additionally, subgroup studies (for different study types) were performed to detect the source of the heterogeneity. The stabilization of study was evaluated by using sensitivity analysis. Publication bias was evaluated by using Begg's test, Egger's test, and funnel plot.

## 3 RESULTS

### 3.1 Study selection and characteristics

**FIGURE 1:**
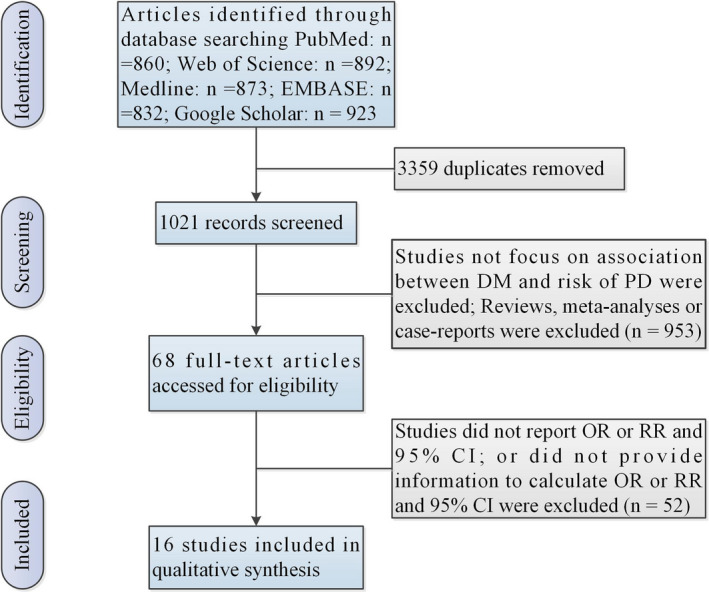
Flow of information through the different phases of a meta‐analysis

Figure 1 shows the selection procedures and finally inclusion results. Table S1 shows characteristics of finally included studies. The present study finally included 7 case–control studies (Becker et al., 2008; D'Amelio et al., 2009; De Pablo‐Fernandez et al., 2017; Miyake et al., 2010; Powers et al., 2006; Schernhammer et al., 2011; Scigliano et al., 2006; including 26,654 PD patients) and 9 cohort studies (De Pablo‐Fernandez et al., 2018; Driver et al., 2008; Hu et al., 2007; Kizza et al., 2019; Palacios et al., 2011; Simon et al., 2007; Sun et al., 2012; Xu et al., 2011; Yang et al., 2017; including 3,819,006 DM patients) exploring the association between DM and risk of PD.

### 3.2 Results of meta‐analysis

**FIGURE 2:**
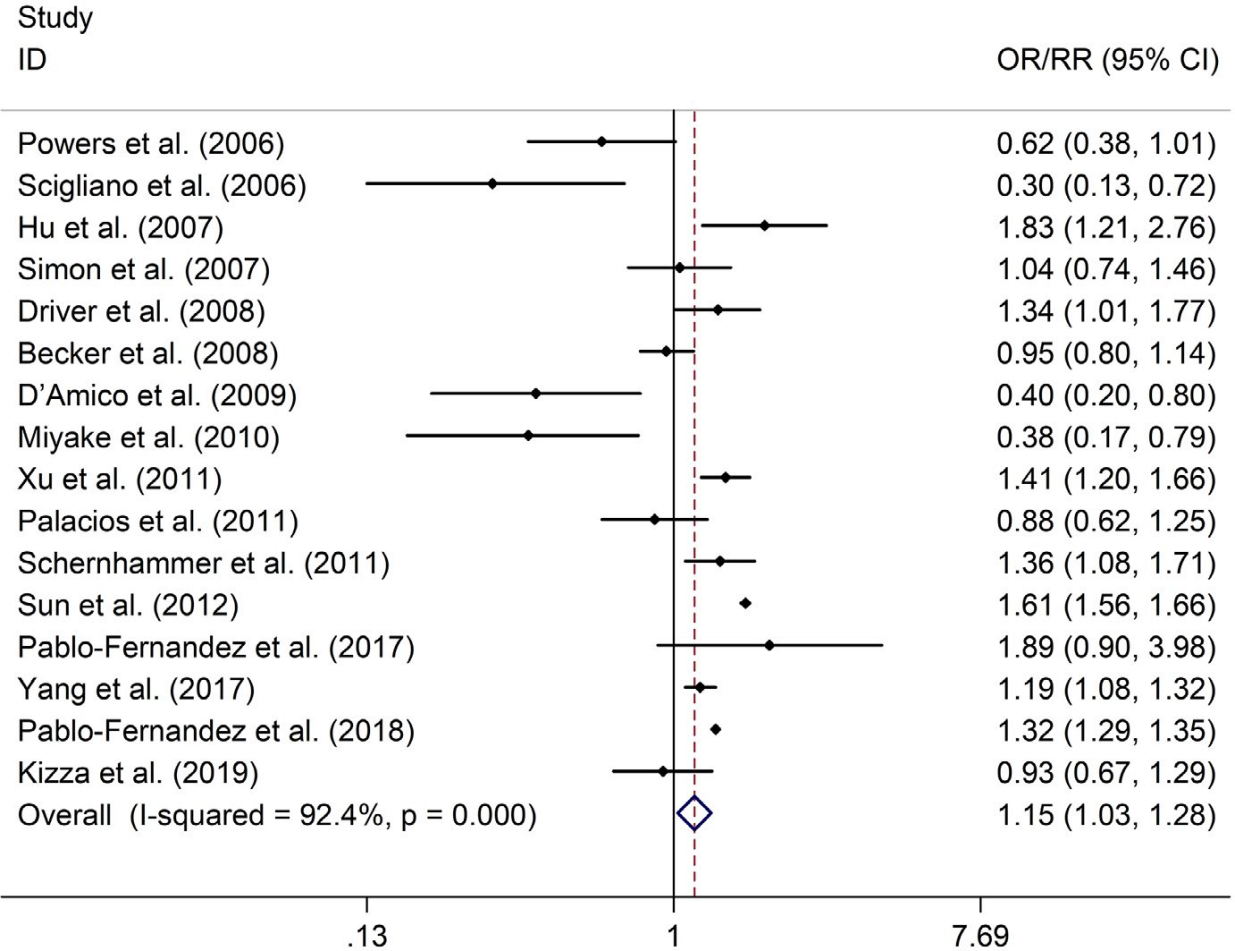
Forest plots of the associations between DM and risk of PD. Abbreviations: CI, confidence interval; DM, diabetes mellitus; OR, odds ratio; PD, Parkinson's disease; RR, relative risk

**FIGURE 3:**
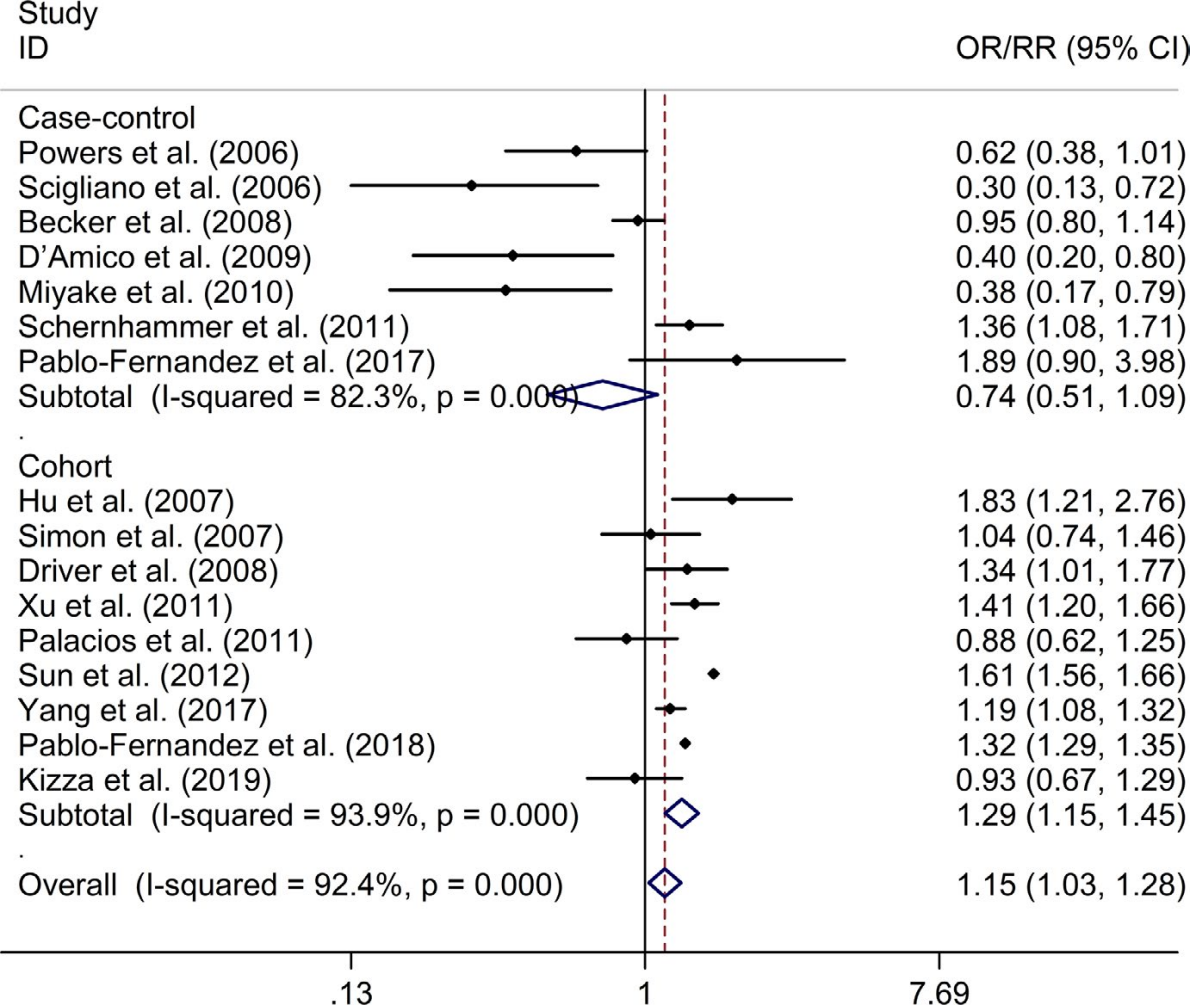
Subgroup study regarding the associations between DM and risk of PD in different types of studies. Abbreviations: CI, confidence interval; DM, diabetes mellitus; OR, odds ratio; PD, Parkinson's disease; RR, relative risk

**FIGURE 4:**
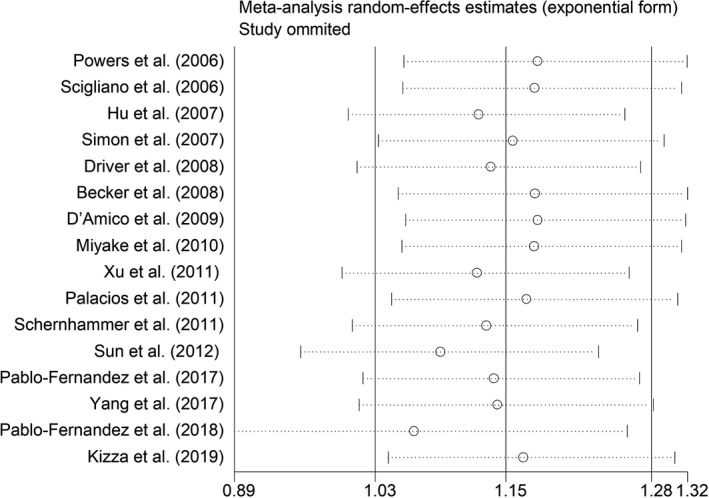
Sensitivity analysis regarding the associations between DM and risk of PD. Abbreviations: CI, confidence interval; DM, diabetes mellitus; OR, odds ratio; PD, Parkinson's disease; RR, relative risk

**FIGURE 5:**
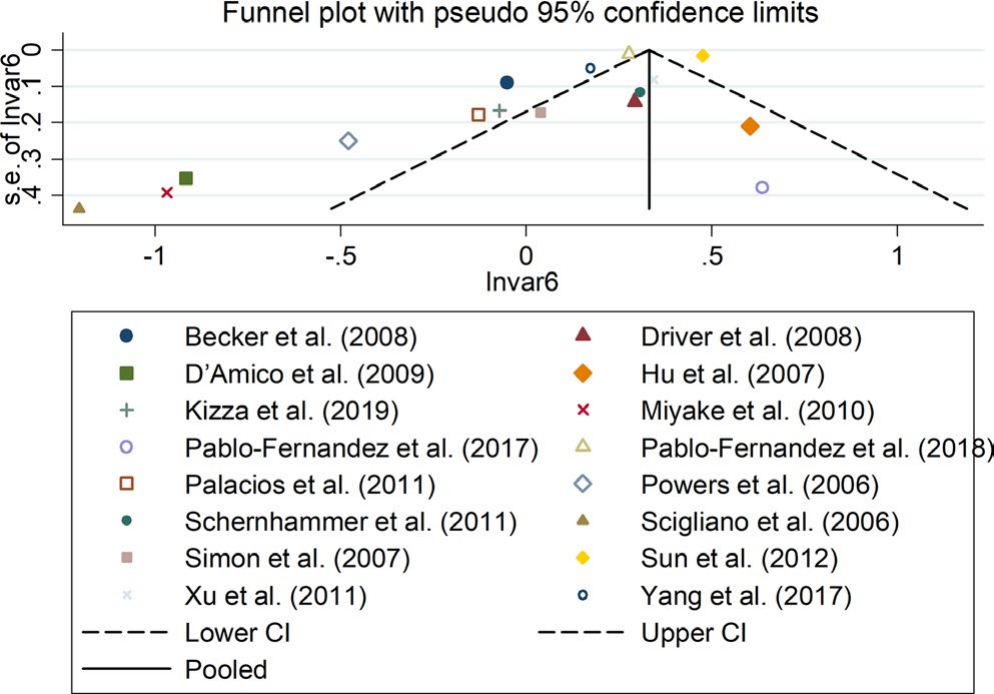
Funnel plots regarding the associations between DM and risk of PD. Abbreviations: CI, confidence interval; DM, diabetes mellitus; OR, odds ratio; PD, Parkinson's disease; RR, relative risk

The meta‐analysis indicated that DM was related to elevated risk of PD (OR/RR = 1.15, 95% CI 1.03–1.28, *I* ^2^ = 92.4%, *p* < .001; Figure 2). Subgroup study showed that DM was associated with higher risk of PD in cohort studies (RR = 1.29, 95% CI 1.15–1.45, *I* ^2^ = 93.9%, *p* < .001; Figure 3), whereas no significant association was indicated between DM and risk of PD in case–control studies (OR = 0.74, 95% CI 0.51–1.09, *I* ^2^ = 82.3%, *p* < .001; Figure 3). Sensitivity analysis showed no changes in the direction of effect when any one study was excluded from all meta‐analyses (Figure 4). In addition, Begg's test, Egger's test, and funnel plot showed no significant risks of publication bias (Begg's test: *p* = .444; Egger's test: *p* = .077; Figure 5).

## 4 DISCUSSION

In this meta‐analysis based on 16 studies analyzing the relationship between DM and PD in over 3,800,000 individuals, we noticed that patients with DM were associated with a 15% increase in the risk of developing PD, compared with patients without DM. In subgroup analyses, cohort studies showed positive correlation between DM and PD, while case–control studies suggested no association.

The potential mechanisms of the role of DM in PD developing have been growing concern. Many risk factors for DM are overlapped with those for PD. Advanced age is a main risk factor for both DM and PD. Meanwhile, both DM and PD are related to chronic inflammation, which performs an important function in the occurrence and development of these diseases (De Virgilio et al., 2016; Lontchi‐Yimagou et al., 2013). Some studies showed that shared dysregulated pathways may lead to DM and PD, such as mitochondrial dysfunction and autophagy (Santiago & Potashkin, 2013).

Our results of the meta‐analysis are consistent with some previous studies (De Pablo‐Fernandez et al., 2018; Yang et al., 2017). The previous systematic review by Cereda et al., (2011) revealed that DM was a risk factor for PD in view of 4 cohort studies; however, it also showed no association between DM and PD based on 5 case–control studies. And result from Lu et al., 2014 was identical to the result of case–control studies conducted by Cereda et al. So we considered that these distinct results may be owing to differences in study design.

Although we included the published literature on the association between DM and PD as much as possible to increase the credibility of analysis results and decrease the risk of bias, some limitations should be noticed. First, the exclusion of unpublished literature may result in a potential bias because of the lack of negative results. Second, we cannot get detailed information related to covariates of PD, such as family history of DM patients, cigarette smoking, and alcohol drinking.

In a word, we have tried to determine whether prior onset of DM may contribute to the risk of developing PD. More and more large‐scale prospective studies should be conducted to evaluate the relationship between DM and PD.

## DISCLOSURE

No conflict of interest.

## AUTHOR CONTRIBUTION

Wei Liu participated in research design, the writing of the paper, the performance of the research, and data analysis. Jianfeng Tang participated in research design and data analysis.

## ETHICAL STATEMENT

The present study was a meta‐analysis. No ethical statement is provided.

### PEER REVIEW

The peer review history for this article is available at https://publons.com/publon/10.1002/brb3.2082.

## Supporting information

Table S1

## Data Availability

The data that support the findings of this study are available on request from the corresponding author. The data are not publicly available due to privacy or ethical restrictions.
