# Supplementary material for: Association between diabetes mellitus and risk of Parkinson's disease: A prisma‐compliant meta‐analysis
Source: Brain Behav. 2021 Jul 21;11(8):e02082. doi: 10.1002/brb3.2082 (PMC8413776; doi:10.1002/brb3.2082)
Supplement: Supplementary file 1 — Table S1 [file BRB3-11-e02082-s001.docx]

Supplementary table 1. Characteristics of included studies.

| References | Study design | Country | Sample Size (male %) | Events for analysis | Adjustment factor | Results |
| --- | --- | --- | --- | --- | --- | --- |
| Hu et al. (2007) ^[^[^1^](#_ENREF_1)^]^ | Cohort | Finland | 51552 (48.8%) | Incidence of Parkinson’s disease | age, study year, BMI , systolic blood pressure, cholesterol, education, leisure-time physical activity, cigarette smoking, coffee consumption, tea consumption, and alcohol consumption | RR: 1.83 (1.21, 2.76) |
| Driver et al. (2008) ^[^[^2^](#_ENREF_2)^]^ | Cohort | USA | 21841 (100%) | Incidence of Parkinson’s disease | age, smoking status, alcohol use, BMI, physical activity vigorous enough to work up a sweat, hypertension, and cholesterol levels | RR: 1.34 (1.01, 1.77) |
| Simon et al. (2007) ^[^[^3^](#_ENREF_3)^]^ | Cohort | USA | 171879 (29.6%) | Incidence of Parkinson’s disease | age and smoking status | RR: 1.04 (0.74, 1.46) |
| Xu et al. (2011) ^[^[^4^](#_ENREF_4)^]^ | Cohort | USA | 288662 (58.2%) | Incidence of Parkinson’s disease | age, sex, race, education, smoking, coffee, BMI, and physical activity | RR: 1.41 (1.20, 1.66) |
| D’Amico et al. (2009) ^[^[^5^](#_ENREF_5)^]^ | Case-control | Italy | 636 (48.1%) | Incidence of Parkinson’s disease | gender, age at PD onset, BMI, smoking habit, alcohol and coffee  consumption | OR: 0.4 (0.2, 0.8) |
| Becker et al. (2008) ^[^[^6^](#_ENREF_6)^]^ | Case-control | UK | 7274 (60%) | Incidence of Parkinson’s disease | BMI, smoking, asthma/COPD, dementia, hypertension, ischemic heart disease, congestive heart failure, stroke/transient ischemic attack, arrhythmia, hyperlipidemia, epilepsy, affective disorders, schizophrenia, and neurotic and somatoform disorders | OR: 0.95 (0.80, 1.14) |
| Miyake et al. (2010) ^[^[^7^](#_ENREF_7)^]^ | Case-control | Japan | 617 (37.9%) | Incidence of Parkinson’s disease | sex, age, region of residence, pack-years of smoking, years of education, leisure-time exercise, body mass index, dietary intake of energy, cholesterol, vitamin E, alcohol, and coffee and the dietary glycemic index | OR: 0.38 (0.17, 0.79) |
| Powers et al. (2006) ^[^[^8^](#_ENREF_8)^]^ | Case-control | USA | 836 (61.6%) | Incidence of Parkinson’s disease | age, ethnicity, education, and smoking | OR: 0.62(0.38, 1.01) |
| Scigliano et al. (2006) ^[^[^9^](#_ENREF_9)^]^ | Case-control | Italy | 711 | Incidence of Parkinson’s disease | age and sex | OR: 0.30 (0.13, 0.72) |
| Schernhammer et al. (2011) ^[^[^10^](#_ENREF_10)^]^ | Case-control | Denmark | 11582 (58.1%) | Incidence of Parkinson’s disease | age, sex, and COPD | OR: 1.36 (1.08, 1.71) |
| Pablo-Fernandez et al. (2018) ^[^[^11^](#_ENREF_11)^]^ | Cohort | UK | 2017115 (53.0%) | Incidence of Parkinson’s disease |  | RR: 1.32 (1.29, 1.35) |
| Pablo-Fernandez et al. (2017) ^[^[^12^](#_ENREF_12)^]^ | Case-control | Spain | 4998 (42.3%) | Incidence of Parkinson’s disease | sex, age, hypertension, dyslipidaemia, antidiabetic treatment, alcohol consumption, smoking status, body mass index, presence of cerebrovascular disease and treatment with potential parkinsonism-inducing drugs | OR: 1.89 (0.90, 3.98) |
| Palacios et al. (2011) ^[^[^13^](#_ENREF_13)^]^ | Cohort | USA | 147096 (43.0%KI) | Incidence of Parkinson’s disease | age, smoking, alcohol intake, caffeine intake, calories, dairy intake, pesticide exposure, physical activity and education | RR: 0.88(0.62, 1.25) |
| Kizza et al. (2019) ^[^[^14^](#_ENREF_14)^]^ | Cohort | China | 503497 (40.8%) | Incidence of Parkinson’s disease | age-at-risk, region, income, education, occupation, alcohol consumption, and physical activity | RR: 0.93(0.67, 1.29) |
| Yang et al. (2017) ^[^[^15^](#_ENREF_15)^]^ | Cohort | China, Taiwan | 145176 (53.4%) | Incidence of Parkinson’s disease | age, gender, insurance premium, urbanization level, residential area, type of occupation, comorbidity, CCI score, flunarizine use, metoclopramide use, zolpidem use, and outpatients claim times | RR: 1.19(1.08, 1.32) |
| Sun et al. (2012) ^[^[^16^](#_ENREF_16)^]^ | Cohort | China, Taiwan | 472188 (49.4%) | Incidence of Parkinson’s disease | age, sex, geographic area, urbanization status, hypertension, hyperlipidemia, and cardiovascular disease | RR: 1.61 (1.56, 1.66) |

Abbreviations: BMI, body mass index; COPD, chronic obstructive pulmonary disease; OR, odds ratio; RR, relative risk; USA, united states; UK, united kingdom.
